# The Roles of Inflammation in Keloid and Hypertrophic Scars

**DOI:** 10.3389/fimmu.2020.603187

**Published:** 2020-12-04

**Authors:** Zheng-Cai Wang, Wan-Yi Zhao, Yangyang Cao, Yan-Qi Liu, Qihang Sun, Peng Shi, Jia-Qin Cai, Xiao Z. Shen, Wei-Qiang Tan

**Affiliations:** ^1^Department of Plastic Surgery, Sir Run Run Shaw Hospital, Zhejiang University School of Medicine, Hangzhou, China; ^2^Department of Physiology, Zhejiang University School of Medicine, Hangzhou, China; ^3^Department of Cardiology of the Second Affiliated Hospital, and Institute of Translational Medicine, Zhejiang University School of Medicine, Hangzhou, China

**Keywords:** immune cells, inflammatory mediators, signal transduction pathways, keloid, potential therapeutics, hypertrophic scar

## Abstract

The underlying mechanisms of wound healing are complex but inflammation is one of the determining factors. Besides its traditional role in combating against infection upon injury, the characteristics and magnitude of inflammation have dramatic impacts on the pathogenesis of scar. Keloids and hypertrophic scars are pathological scars that result from aberrant wound healing. They are characterized by continuous local inflammation and excessive collagen deposition. In this review, we aim at discussing how dysregulated inflammation contributes to the pathogenesis of scar formation. Immune cells, soluble inflammatory mediators, and the related intracellular signal transduction pathways are our three subtopics encompassing the events occurring in inflammation associated with scar formation. In the end, we enumerate the current and potential medicines and therapeutics for suppressing inflammation and limiting progression to scar. Understanding the initiation, progression, and resolution of inflammation will provide insights into the mechanisms of scar formation and is useful for developing effective treatments.

## Introduction

Wound healing is one of the most common events repeatedly occurring through an entire human life. Nevertheless, it is a complex process comprising five phases, i.e., hemostasis, inflammation, proliferation, re-epithelialization, and remodeling (1). After injury, hemostasis is immediately triggered with cascades of reactions of platelet plug and clot formation for arrest of bleeding. In the second step, damaged tissues, together with activated platelets, arouse inflammatory responses by recruiting immune cells such as neutrophils and macrophages. When inflammation subsides, new blood vessels and connective tissues appear, marking the beginning of a proliferation stage in which wound area also shrinks due to wound contraction. A conductive event of proliferation is re-epithelialization which is mainly driven by keratinocytes migration. After that, tissue maturation will be the hallmark of the remodeling stage; this stage involves regression of neovasculature and a concomitant reconstitution of extracellular matrix (ECM), resulting in organized collagen fibrils which are the basis of normal scar. Each stage of wound healing requires intricate synchronization and regulation of multiple cellular populations. Perturbations to any of these processes might lead to a spectrum ranging from nonhealing wounds to excessive scarring, including hypertrophic scars and keloid. The excessive scarring is characterized by disorganized and redundant deposition of ECM resulting from abnormal proliferation and differentiation of fibroblasts. Excessive scarring has many adverse consequences including disfiguring, pain, itching, contracture, and motion restriction, inflicting the injured both physically and psychologically. The difference between hypertrophic scars and keloid lies in prognosis, with the former developing within the original wound boundaries and prone to regress over time, while the latter growing without limitation and rarely regressing (1).

The mechanisms of formation of hypertrophic scar and keloids have not yet been completely understood. However, many studies have shown that inflammation is involved in modulating collagen synthesis, and the intensity of inflammation is positively correlated to final scar sizes (2–4). In this review, we stratify inflammation to three levels of players, i.e., inflammatory cells, inflammatory mediators, and the general signal pathways involved in inflammation, and discuss their potential roles in keloid and hypertrophic scar formation. In the end, we also summarized currently available treatments and potential therapeutic strategies targeting on inflammation for suppressing scar formation.

## Inflammatory Cells

Inflammation takes place right after skin wounding due to tissue damage and microbial invasion. Both skin tissue cells and immune cells are equipped with pattern recognition receptors (PRRs) which are conserved among species for detecting pathogen-associated molecular patterns (PAMPs) and damage-associated molecular patterns (DAMPs). PAMPs are derived from microorganisms. These patterns are alien to the hosts and are found on bacterial cell walls, DNA, lipoproteins, carbohydrates, or other structures, and drive inflammation through PRRs. Concurrently, upon injury, wounding activated DAMPs, a plethora of endogenous molecules, that are abnormally released by damaged or stressed cells (5). Many metabolites can act as DAMPs (6), including adenosine 5′-triphosphate (ATP), uric acid and oxidized low-density lipoprotein (oxLDL). DAMPs can also be proteins including HMGB1, S100, and heat shock proteins (HSPs). They are sensed by either PRRs or their specific receptors (for example, P2 receptors to ATP). When tissue is damaged or under stress, DAMPs may be released or increasingly generated from cells, and the elevated extracellular concentrations of DAMPs can mobilize and activate immune cells.

**Figure 1:**
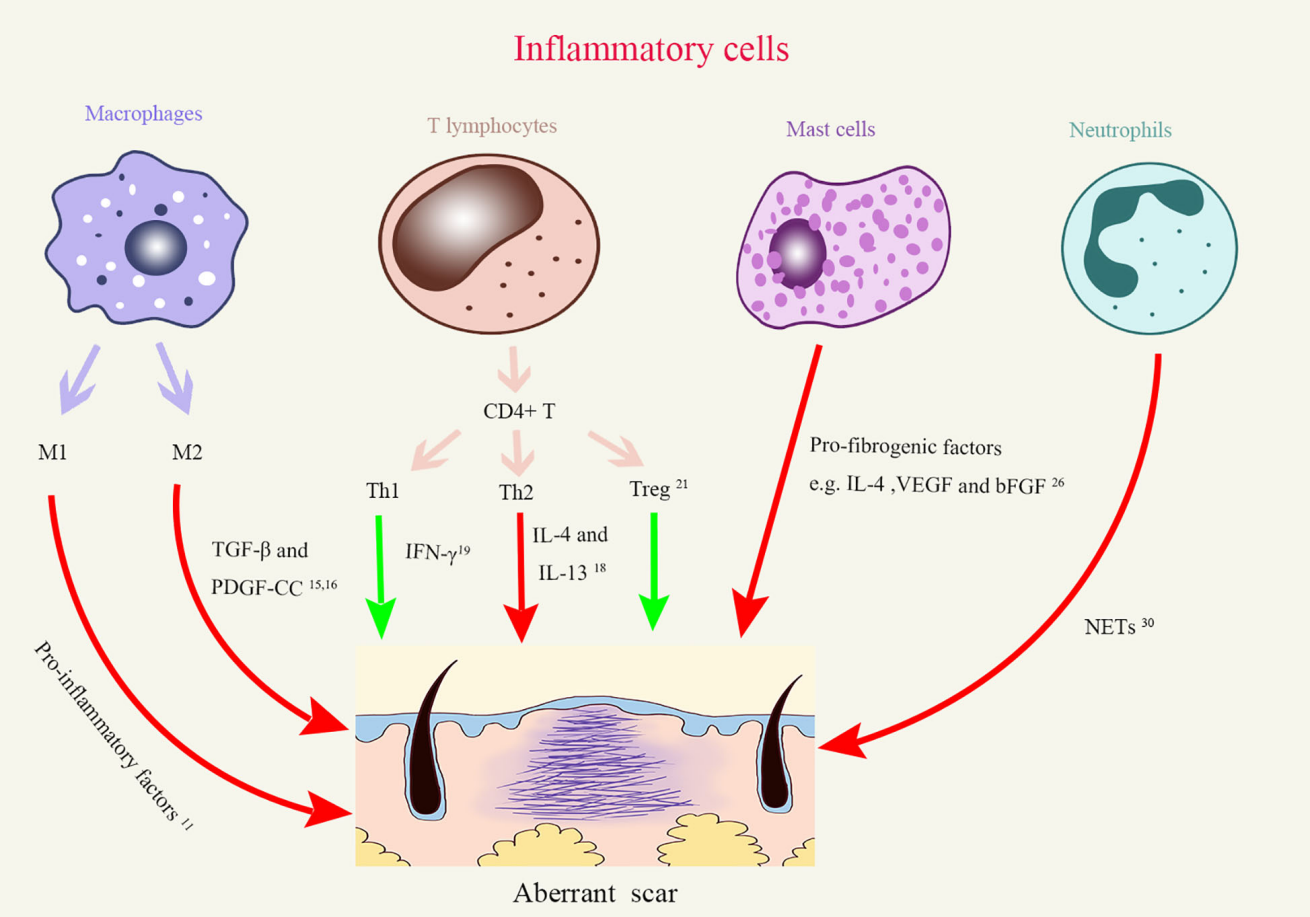
The roles of different inflammatory cells in aberrant scar formation. Red arrows, Positive effects; Green arrows, Negative effects; Treg, regulatory T cells; Th, Helper T cells; TGF-β, Transforming growth factor-β; PDGF-CC, Platelet-derived growth factor–CC; IFN-γ, Interferon γ; IL-4, interleukins-4; IL-13, interleukins-13; VEGF, Vascular endothelial growth factor; bFGF, basic fibroblast growth factor; NETs, Neutrophil extracellular traps.

Inflammatory cells are the executers of inflammatory responses. During the inflammation phase of a typical wound healing process, immune cells are thought to be mainly recruited for preventing the invasion of pathogenic microbes. However, dysregulation of immune cells would alter the outcome of wound healing and lead to aberrant scaring. In fact, macrophages, lymphocytes, mast cells, and neutrophils have been separately reported to be involved in the development of scar (**Figure 1**). In particular, macrophages, T cells, and mast cells all increased in keloid tissue, although in varying degrees (7, 8). As we discuss below, these cells can regulate different aspects of fibrotic processes.

### Macrophages

Macrophages are a major player in tissue remodeling after damages (9–11). Macrophages undergo M1 (classical) or M2 (alternative) activation, which represent extremes in a continuum of activation states. The current prevailing opinion is that M1 macrophages but not their M2 counterparts overwhelmingly exist in the tissues around the wound in the inflammation stage, in that they secrete a plethora of proinflammatory cytokines. In contrast, later in the proliferation, re-epithelialization and remodeling stages, the tissues around the wound mainly contain M2 macrophages which promote angiogenesis and collagen deposition. The M1 macrophages come from bone marrow-derived monocytes, as MCP-1 (CCL2), a chemokine essential for monocyte egress from the bone marrow, is critical for the accumulation of M1 macrophages in wounds. The origin of M2 macrophages is not yet clarified and may have multiple sources. One study documented that M1 cells can transform to M2 cells once they phagocytose neutrophils during wound healing (12), indicating that reprogramming takes place in the local tissues at the phase of clearance. Actually, the late stages of wound healing have many similarities to tumor growth where cell proliferation and vasculature formation are actively taking place. The macrophages in tumor tissues generally have M2 phenotype induced by tumor-derived soluble factors such as GM-CSF and IL-6. Thus, contributions of microenvironmental cues cannot be excluded to the transition of macrophages, and these cues may also drive the differentiation of newly arrived monocytes to M2 macrophages.

In keloid tissue, macrophages upregulated M2-associated genes which are highly relevant to tissue repair and remodeling (13). It is thus unsurprising that macrophage overabundance was correlated with abnormal scar formation (14). These macrophages could promote the transformation of fibroblasts into myofibroblasts by secreting transforming growth factor-β (TGF-β) and platelet-derived growth factor–CC (PDGF-CC), both of which facilitate collagen deposition and scar formation (15, 16). These observations made in mouse studies were further confirmed in patients with hypertrophic scars, who had significantly more macrophages of M2 phenotype in their skin even before the occurrence of trauma than individuals who had normal scars (17). These results suggest a promoting role of M2 macrophages in fibrosis. However, a definite cause-and-effect relationship is still lacking.

An elegant study performed by Lucas et al. demonstrated that depletion of macrophages at different stages had distinctive impacts on wound healing (11). By using an LysMCre/iDTR mouse model, conditional depletion is plausible. Mice benefited from macrophages depletion in the inflammatory stage, as manifested by reduced vascularized granulation tissue and minimized scar formation. This is consistent with the observation that extended inflammation phase correlates with excessive scar formation (14). In contrast, depletion of macrophages in the proliferation and re-epithelialization stages resulted in severe hemorrhage in the wound tissue, implying important role of macrophages in re-establishing skin integrity. Intriguingly, depletion of macrophages in the remodeling stage had limited impact on the degree of fibrosis and scar formation, which is contradictory to the current consensus that macrophages in the remodeling phase promote scar formation. However, this study was performed in mice which usually have very limited scar formation after skin wounding. Thus, studies with proper models for pathological scar formation, such as rabbit ear hypertrophic scar model or keloid scar implantation model, should be performed to validate the roles of macrophages in the remodeling phase. Nevertheless, this study clearly indicates that early interfering macrophage accumulation is beneficial to wound healing.

### T Lymphocytes

Current research revealed complex roles of T cells in regulating scar formation, partly due to diversity of T cell subsets. In addition to a dichotomy between CD8^+^ cytotoxic T cells and CD4^+^ helper T cells, CD4^+^ T cells can be further subgrouped to Th1, Th2, Th17, Tfh, and regulatory T (Treg) subsets based on transcriptomes and effector cytokines. The interleukins-4 (IL-4) and IL-13 secreted by Th2 cells are suggested by many fibrotic models to promote the synthesis and metabolism of collagen, resulting in reticular fibrin deposition (18). Th1 cells, on the other hand, attenuated tissue fibrosis through secreting IFN-γ (19) which is capable of suppressing fibroblast proliferation and downregulating type I, III collagen gene expression (20). It is known that Treg are capable of inhibiting other effector T cells and maintaining peripheral immune tolerance. This immunosuppressive function has a direct impact on collagen deposition in scar formation. *In vitro*, coexistence of Treg matters in collagen secretion by fibroblasts. Coculture of keloid fibroblasts with a Treg-enriched condition (approximate 35% Tregs in a mixed T cell population) reduced collagen synthesis in comparison to the keloid fibroblasts cocultured with a Treg-deficient T cell population (21). Interestingly, without other CD4^+^ T cells, coculture of fibroblasts with purified Treg cells actually enhanced expression of collagen by the former (22). The disparity of the experiments talked above indicates that besides a direct regulatory role of Tregs on fibroblasts, interaction between Tregs with other T helper cells can impart an indirect modulation of collagen deposition and the subsequent scar formation process by Treg cells. Superimposing on this complexity, it is also suggested that CD4^+^ T cells preconditioned in different microenvironment have different effects on scar formation. When compared to the CD4^+^ lymphocytes derived from normal subjects, CD4^+^ T cells derived from post-burn patients who tended to form hypertrophic scars promoted fibroblast proliferation and collagen synthesis *in vitro* (23).

Altogether, research so far is yet conclusive regarding the roles of T cell subtypes in scar formation. The timing and duration of inflammation is critical for the final outcome of scar formation. However, delineation of the kinetics of infiltration of Th cell subtypes into skin wound still lacks, partly due to deficiency of right tools to monitor them in situ. Even the antifibrotic/profibrotic Th1/Th2 dogma requires an exploration for confirmation in skin scar models.

### Mast Cells

Mast cells, the essential cell type in allergic responses, have been gradually appreciated to be participants in chronic non-allergic inflammation as well (24). For example, their number significantly increased in keloid (25), indicating a link between mast cells and aberrant scar formation. Evidence supporting this notion came from the observation that the numbers and activation status of mast cells were positively correlated with the degrees of scars (26). To explain the mechanisms, the authors performed coculture experiments and demonstrated that mast cells could stimulate fibroblast proliferation through releasing IL-4, VEGF, and basic fibroblast growth factor (bFGF) (26), resulting in increased type I collagen synthesis. This effect of upregulation of collagen expression could be abrogated by blocking PI-3K/AkT signaling pathway in mast cells (27).

It was noticed that in mice, wounds generated at embryonic day 15 (E15) healed without leaving a scar, whereas those generated at embryonic day 18 (E18) healed with a scar (REF). Wulff et al. believed that the developmental status of mast cells may explain this difference (28). Mast cells at E15, compared with those at E18, were fewer in number, were less mature and failed to degranulate after wounding. The scarless wound healing process at E15 could be disrupted by injecting mast cell lysates immediately after wounding, corroborating an underdeveloped phenotype of mast cells at E15. In contrast, wounds produced at E18 healed with significantly smaller scars in mast cell-deficient Kit^W/W-v^ mice compared with their Kit^+/+^ littermates, suggesting a determinant role of mature mast cells in scar formation. However, controversy still exists. A comprehensive study performed by Nauta et al. showed a dispensable role of mast cells in adult would healing (29). By using three mast cell-deficient mouse lines C57BL/6-Kit^W-sh/W-sh^, WBB6F1-Kit^W/W-v^, and Cpa3-Cre: Mcl-1^fl/fl^ mice, the authors concluded that mast cells did not play a significant role in the healing of splinted full thickness excisional cutaneous wounds in adult mice. Thus, mast cells appear involved in embryo scar formation but are redundant in wound healing of adult mice.

### Neutrophils

Neutrophils are the earliest leukocytes to arrive on the loci of injury. They are the key cells preventing microbes spreading. One of the major sterilization patterns exerted by neutrophils is the formation of neutrophil extracellular traps (NETs). By releasing chromatin, histones, and MPO, these NET structures can not only trap and kill microbes, but also can promote the differentiation of human lung fibroblasts and their fibrosis activity, at least *in vitro* (30). Supplemented with NETs, myofibroblasts expressed more connective tissue growth factors and collagen fibers. These pro-fibrotic effects were impeded by degradation of NETs with DNase1, heparin or myeloperoxidase inhibitor, suggesting rather the NET structure *per se* than the individual components of NETs matters in fibrotic process (30). Although the effect of neutropenia on wound closure was briefly investigated (30), *in vivo* evidence of neutrophils’ roles in scar formation is lacking. Given the importance of activated neutrophils in producing oxidative species and recruiting monocytes, more work is needed to elaborate their roles in scarring.

## Inflammatory Cytokines

**Figure 2:**
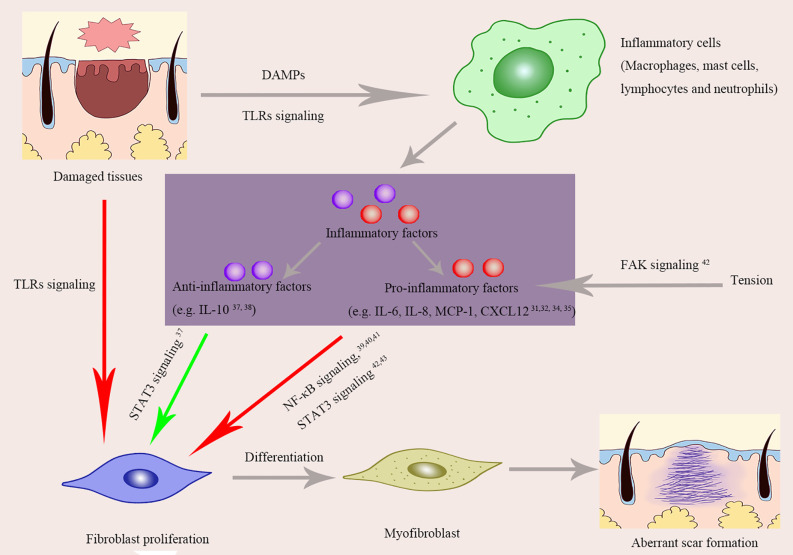
The roles of inflammatory factors and inflammatory signaling pathways in aberrant scar formation. Red arrows, Positive effects; Green arrows, Negative effects; TLRs, Toll-Like Receptors; DAMPs, damage associated molecular patterns; NF-κB, Nuclear factor kappa B (NF-κB); STAT-3, Signal transducer and activator of transcription 3; FAK, Focal adhesion kinase; IL-6, Interleukin-6; IL-8, Interleukin-8; MCP-1, Monocyte chemoattractant protein-1; CXCL12, Chemokines ligand 12.

In many occasions, cytokines/chemokines are the essential mediators of inflammatory cells to exert their roles. The fact that a certain cytokine may have distinctive effects based on microenvironments places another dimension of complexity for understanding the roles of cytokines in inflammation and tissue remodeling. In the early stages of wound healing, inflammatory factors usually display pro-inflammatory and they are orchestrated to make defense mechanisms in action. In contrast, cytokines with anti-inflammatory effects overwhelm in the late stages to promote tissue cell proliferation and remodeling. Imbalance of cytokines in play at any stage of wound healing may lead to aberrant scar formation (**Figure 2**). In a general consensus for now, excessive and prolonged pro-inflammatory reaction is favorable to the occurrence of pathological scars.

### Pro-Inflammatory Effects and Scar Formation

A plethora of factors including IL-6, IL-8, IL-18, chemokine like factor-1 (CKLF-1), prostaglandin produced by cyclooxygenase (COX-1) that exhibit pro-inflammatory roles upon tissue damage have been found significantly elevated in keloid tissue (31, 32). Even in the peripheral blood of keloid patients, IL-8 level was 7 fold higher than that of normal people (33). However, it is unclear that the rise of these soluble molecules is cause or consequence of keloid formation. Many studies so far had tried to identify the cause-and-effect relationship between these molecules and scar formation in animal models. Artificial manipulation of the expression of a certain cytokine is a common way for interrogating the role of a specific molecule. By both over-expression and genetic depletion, Nishiguchi et al (34). revealed that chemokine CXCL12 could promote scar formation in mice: CXCL12 induction led to lager scar size, whereas CXCL12 abrogation downregulated scar formation. Similar observation was made for MCP-1. Even after bleomycin injection, mice with MCP-1 deficiency had milder inflammation and a similar architecture to normal skin, while wild-type mice went through robust inflammation and had thickened collagen bundles with abnormal arrangement (35). IL-17, a prototypic cytokine of Th17 cells, was found elevated in hypertrophic scar tissues. Injection of recombinant IL-17 to wound area at the inflammatory stage resulted in aggravated fibrogenesis and increased inflammation with higher levels of MCP-1, MCP-2, and MCP-3. This pro-fibrotic effect of IL-17 could, however, be blocked by depleting macrophages with clodronate liposomes, suggesting that macrophages mediate the pro-fibrotic effect of IL-17 (36).

### Anti-Inflammatory Effects and Scar Formation

Molecules that mainly display anti-inflammatory effects on would healing are generally thought to be beneficial for preventing deformed scarring. For example, IL-10 has been shown to negatively regulate collagen synthesis, eventually reducing scar formation. This anti-fibrotic effect was abrogated by blockade of IL-10 receptor or its downstream PI3k/Akt pathways (37). The anti-inflammatory and anti-scar effects of IL-10 were also verified in gene knockout animal. Mice deficient of both IL-10 and IL-4 showed exaggerated inflammatory responses and deteriorated scar formation. By contrast, supplementation of IL-10 to wound area resulted in alleviated scar formation, which was observed in both animal model and a human study (38).

## Signaling Pathways Linking Inflammatory to Pathological Scars

Upon binding with their cognate ligands, cytokine receptors change their configurations and trigger downstream signaling pathways. Many cytokine receptors are heterodimers and share chains, and moreover, some downstream signal pathways could be activated by multiple receptors. Herein, we discuss a couple of key pathways relevant in scar formation (**Figure 2**).

### Nuclear Factor Kappa B (NF-κB)

The NF-κB family members are themselves transcription factors that regulate many key inflammatory genes. The NF-κB pathway was demonstrated to be activated in keloid fibroblast (39, 40). Specifically, 15 genes downstream of NF-κB signaling were up-regulated in keloid fibroblasts compared to normal skin fibroblasts after TNF-α treatment. Even in the baseline, the protein level of NF-κB and NF-κB-binding activity were higher in keloid fibroblasts (39). Blocking this pathway with dehydroxymethylepoxyquinomicin (DHMEQ) resulted in decreased proliferation of fibroblasts and less deposition of type I collagen, implying that NF-κB pathway is involved in keloid pathogenesis (40). This notion was further supported by the study of Fujita et al. (41) who identified that one of the transcript variants of NEDD4 gene was highly associated with keloid formation in that it could activate NF-κB signaling pathway in keratinocytes and fibroblasts, confirming the essential role of NF-κB pathway in the development of keloid.

### STAT-3

The STAT-3 signaling pathway is downstream of a variety of cytokines and acts as a regulator of cell proliferation, migration, differentiation, apoptosis, inflammation, as well as fibrosis. In keloid tissues, this signaling pathway was activated (41, 42). Decreasing the expression of STAT-3 or inhibiting its phosphorylation could significantly reduce synthesis of collagen and proliferation and migration of keloid fibroblasts (42), supporting a pro-fibrotic role of STAT-3. Besides keloid fibroblasts, a pro-fibrotic role of STAT-3 pathway was also confirmed in hypertrophic scar fibroblasts. With an IL-6•IL-6Rα complex treatment, STAT-3 pathway was activated in fibroblast from hypertrophic scar, leading to upregulation of procollagen (Col1A2), fibronectin 1, and the cellular proliferation marker (c-Myc), which could be reverted by STAT-3 suppression. This study implicates a positive role of STAT-3 pathway in mediating IL-6 signaling for scar formation (43).

### Focal Adhesion Kinase

High local mechanical force has been implicated in the development of abnormal skin fibrosis. This was established by clinical observation that keloid and hypertrophic scar tend to locating on anterior chest and scapular regions where constant tension is present. The tyrosine kinase FAK (focal adhesion kinase) is known for mechano-transduction. It is thus not surprising to observe an elevated FAK signaling in keloid tissues (44). To explore its specific role, Wong et al. carried out an elegant and systematic research, showing that FAK signaling induced by mechanical force accelerated skin fibrosis via promoting inflammation (45). By using fibroblast-specific FAK knockout mice and MCP-1 knockout mice, they convincibly concluded that mechanical force could affect fibrosis through the inflammatory FAK–ERK–MCP-1 pathway (45).

## Targeting Inflammation to Prevent and Treat Pathological Scars

**Figure 3:**
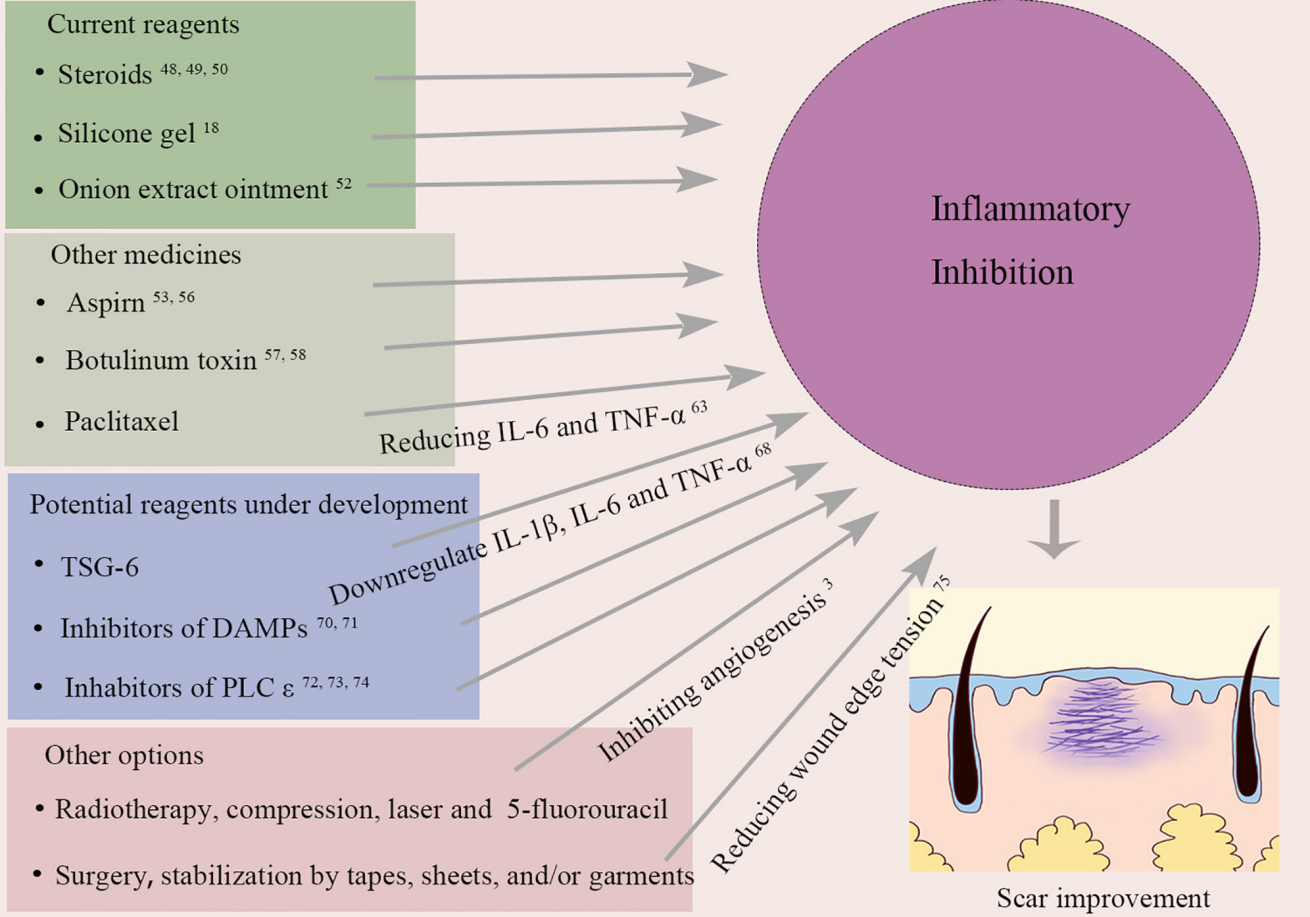
Current and potential treatments for pathological scar through suppressing inflammation. IL-6, Interleukin-6; TNF-α, tumor necrosis factor-α; TSG-6, tumor necrosis factor alpha stimulated gene-6; IL-1β, Interleukin-1β; DAMPs, damage associated molecular patterns; PLC ϵ, phospholipase ϵ.

Many reagents have been reported effective in patients or animal models on suppressing scar formation, which is more or less related to their anti-inflammatory effects. Herein, we will discuss the reagents clinically available and those under development as well (**Figure 3**). Some of them were initially designed for other purposes but later were found applicable to scar treatment. It is worth noting that animal data should be translated with extra caution since human wound healing mainly occurs through re-epithelialization while skin contraction plays a main role in the early stage of wound healing of other mammalians such as mouse.

### Current Reagents Used in Scar Management

Steroids are well known for their anti-inflammatory effect and are widely used in treatment for autoimmune diseases. They decrease inflammation by suppressing the activities of both myeloid and lymphoid cells (46). In agreement with the opinion that extravagant inflammation promotes excessive scars, steroid administration is one of the most effective treatments for keloid and hypertrophic scars (47). For example, intradermal injection of triamcinolone acetonide, as one of recommended choice for the treatment of keloids, has reported the best success rates for scar treatment (48, 49). The underlying mechanism of it may attributed to its anti-inflammation effects. Oxandrolone, which is usually prescribed for wasting diseases, has recently been found to be anti-fibrotic in rabbit ear hypertrophic scar model (50). Administration of oxandrolone could decrease inflammation in the wounds, inhibit the activity of fibroblasts and myofibroblasts, reduce the deposition of collagen, and prevent the formation of hypertrophic scars. This scar-ameliorating effect of oxandrolone was further confirmed by a randomized clinical study that found significant improvement of scar and pliability in children after burn injury (51).

For the last 30 years, silicone gel is considered the first line of treatment for most scar management cases, including hypertrophic and keloid scars. This anti-scar activity could be partly attributed to the hydrated and occlusive environment created by silicone gel dressings, and hydration is also believed to lead to the stabilization of mast cells (18).

Composed of phenolic compounds, onion extract ointment is another common choice for scar treatment. The active ingredient from onion extract is allium cepa which could be converted to quercetin, an anti-inflammatory derivative. Its effects include mast cell stabilization and anti-proliferative effects (52).

### Other Medicines Having Anti-Scar Functions

Aspirin, also known as acetylsalicylic acid, is a widely used nonsteroidal anti-inflammatory drug, NSAID, which can reduce inflammation by inhibiting prostaglandin production. Many studies have confirmed its anti-fibrotic effects. *In vitro*, aspirin could inhibit TNF-α–induced activation of NF-κB (53) and block fibroblast proliferation (54, 55). This is consistent with *in vivo* observations that aspirin could inhibit tendon scar formation after injury through limiting inflammation via regulation of JNK/STAT-3 signaling (56). On the other hand, the ratio of tissue inhibitor of matrix metalloproteinase-3 (TIMP‐3) to matrix metalloproteinase-3 (MMP‐3) could be reduced by aspirin, leading to ECM degradation (56).

Botox is the commercial name of botulinum toxin (BTX), a neurotoxin produced by *Clostridium botulinum*. BTX can inhibit the release of acetylcholine vesicles at the neuromuscular junction so as to block the impulse transmission, resulting in muscle paralysis. It is widely used in cosmetology to reduce wrinkles, suppress masseter hypertrophy, and improve facial contour, etc. Apart from blocking acetylcholine release, BTX has anti-inflammatory effects on wound healing and scar formation (57, 58). A meta-analysis evaluating intralesional injection of BTX A versus corticosteroids and placebo in the treatment of hypertrophic scar and keloid showed that BTX A was more effective than the others (59). Another study conducted by An et al compared the effects of inflammation intervention in earlier phases (operation-day) with that in later phase (2-week postoperative) on scar formation after thyroidectomy (60). Although no difference was observed in scar size, early application of BTX A achieved better appearance of scar in terms of erythema, skin elasticity, and patient satisfaction, compared to its later application.

Paclitaxel (PTX) is an anti-cancer drug which can induce carcinoma cell apoptosis. It has recently been shown to regulate inflammatory responses and fibrosis. For instance, PTX was found to inhibit NF-κB pathway (61) and attenuate interstitial fibrosis in mice kidneys by blocking STAT-3 signaling (62). This anti-inflammatory and anti-fibrosis effect was also observed in animal models of keloid (63) and hypertrophic scar (64). By using keloid-bearing nude mice model, Wang et al. demonstrated PTX had inhibitory effect on keloids growth (63). *In vitro*, the authors were able to show that the expression of IL-6 and TNF-α, as well as the production of α-SMA and collagen I, by human keloids fibroblast decreased after paclitaxel administration (63). This is consistent with the conclusion drawn on a rabbit ear hypertrophic scar model. With paclitaxel treatment, fibroblast proliferation, collagen deposition, and micro-angiogenesis in hypertrophic scars were all downregulated (64).

### Potential Reagents

TSG-6 (Tumor Necrosis Factor, Alpha-Stimulated Gene-6 Protein)

TSG-6 is a secreted 35-kDa glycoprotein that contains multiple adjacent functional domains. TSG-6 is not a constitutively expressed protein but is rapidly upregulated in both inflammatory cells and tissue cells upon exposure to inflammatory mediators, including IL-1β, IFN-γ, TNF-α, LPS, and PGE2 (65). Besides, it can also be induced by many growth factors including FGF, EGF, and TGF-β. Although the specific receptor for TSG-6 has not been identified, this glycoprotein binds to various proteoglycans and polysaccharides, including hyaluronic acid and chondroitin sulfate (66). These interactions primarily act to stabilize or remodel the ECM, so that it could inhibit inflammatory responses by repressing neutrophil migration and modulating the protease network. Analysis of clinical samples revealed that the level of TSG-6 in keloid was significantly lower than that in normal skin (67), implying an inverse correlation between TSG-6 level and degree of keloid. Indeed, in different animal models, administration of recombinant TSG-6 could reduce tissue fibrosis (68, 69). Mechanistically, TSG-6 could reduce the ratio of TGF-β1/TGF-β3 (69) and downregulate the expression of collagen, myeloperoxidase (MPO) and inflammatory molecules IL-1β, IL-6, TNF-α (68). Thus, TSG-6 has showed great potential for clinical application in scar treatment.

#### Inhibitors of DAMPs

High-mobility group box 1 (HMGB1) protein, when released, has been shown to promote inflammation in many occasions including sepsis. Upon skin injury, it could drive infiltration of inflammatory cells and increase collagen synthesis by fibroblasts. HMGB1 secreted from keratinocytes activated fibroblasts by promoting the nuclear import of MRTF-A, increased the nuclear accumulation of MRTF-A/SRF complexes and consequently enhanced α-smooth muscle actin promoter activation (70). Application of HMGB1 inhibitor BoxA significantly suppressed scar formation in rabbit (70). In another study, blockade of HMGB1 production by polydeoxyribonucleotide also reduced inflammation and improved scarring outcomes in rats. Another DAMP molecule, S100A12, has also been shown profibrotic. Intradermal injecting S100A12 increased hypertrophic scar in a rabbit ear model. By contrast, blocking its receptors resulted in inhibition of fibroblast activation (71). Altogether, these studies indicate potential benefit for alleviating scar formation by targeting DAMPs molecules and their pathways.

#### PLC ϵ Blockers

As an effective molecule of Ras/rap small G protein, PLC ϵ activation could modulate skin inflammatory responses by inducing the release of inflammatory factors from fibroblasts and keratinocytes (72). This pathway was verified in PLC ϵ gene overexpressed and deficient mice (73, 74). Mice overexpressing PLC ϵ in keratinocytes exhibited increased expression of IL-23 and aberrant infiltration of T cells (73). By contrast, depletion of PLC ϵ in mice was shown to attenuate scar formation which may result from a decrease of expression of IL-6 and chemokines CXCL-1, CXCL-2, and CCL20, Col6a1 (74). Thus, inhibition of PLC ϵ could be a potential strategy in suppressing scar formation.

### Other Options

Ogawa et al. believed that other treatments, such as radiotherapy, compression (pressure therapy), laser and even 5-fluorouracil (5-FU) therapy could reduce inflammatory by inhibiting angiogenesis, given the fact that inflammatory cells migrate through blood (3). Also, surgery and stabilization by tapes, sheets, and/or garments are considered promising in decreasing inflammation in the wound area by reducing the tension of wound edge (75). In fact, most existing treatments for scarring are more or less associated with their effects in inflammation suppression.

## Conclusions and Future Directions

Normal wound healing requires inflammation and tissue remodeling mounting to appropriate degrees. Excessiveness of either of these two factors leads to the development of keloid or hypertrophic scars. Especially, overabundance of inflammatory cells in the remodeling phase correlates with pathological scar formation, indicating presence of a prolonged inflammation stage overlapping with the later stages. In the process of inflammation, immune cells and soluble inflammatory mediators affect aspects of fibroblast biology in respect to proliferation, differentiation and eventually collagen deposition. Accordingly, a variety of anti-inflammation treatments have been proposed to improve scar outcome. However, most of them have been only evaluated in the remodeling phase of wound healing because 1. pathological scar becomes more obvious on appearance at this stage and 2. excessive inflammation at this stage correlates to severity of scar formation. In contrast, investigation and practice of anti-inflammatory treatments at earlier stages have been very limited, partly due to the assumption that moderate inflammation in early stage is beneficial for wound healing, given the facts that inflammation is critical in preventing infection and promoting neovasculature development. Actually, a few studies had tried to explore the effects of early intervention of inflammation on scar formation. The outcomes varied in different approaches. Early macrophage depletion showed benefits in minimizing scar formation (11), while BTX A treatment improved scar quality but had negligible effect on scar size (60, 76). Thus, more specific early intervention strategies, e.g., suppressing macrophage accumulation, rather than a general anti-inflammation treatment, may be more promising. To this end, extensive investigations should be pursued to address the roles of individual players (immune cells and inflammatory mediators) in wound healing and scar formation.

Current anti-scarring treatments mainly aim on two mechanisms: anti-inflammation and anti-collagen synthesis. As mentioned above, the scar-limiting effect of anti-inflammation treatment is partially mediated through anti-collagen deposition. However, extended inflammation delays wound healing, a pathological-scar inducing factor independent of fibrosis. Thus, combination therapy is recommended to exploit both directions for therapeutic benefits. Optimal therapeutic interventions may aim to balance the extent of inflammation and tissue remodeling. A combined strategy, for example, by sequential application of corticosteroids, onion extract ointment and silicone gel may suit for this aim (77).

Scarless healing is the ultimate goal of scar prevention. To develop an optimal combination strategy, comprehensive cause-effect relationships of key cells and molecules in scar pathogenesis should be delineated, therefore providing novel therapeutic target for scarless healing. High throughput single cell RNA-sequencing may provide profound insights. Moreover, the intriguing possibility emerges that selectively blocking certain pathways in the early stage of wound healing may have prophylactic effects against pathological scar.

## Author Contributions

WQT and XZS conceived the topic. ZCW and XZS wrote the manuscript. PS, WYZ, and JQC contributed to **Figures 1**–**3**. YC, YQL and QS contributed to the revision. All authors contributed to the article and approved the submitted version.

## Funding

This work was supported by grants from National Natural Science Foundation of China (No. 31970898, 81670378, 81700365, 31771266, 81701911, 81671918), National Key Research Program of China (2016YFC1101004) and Zhejiang Provincial Medical and Healthy Science Foundation of China (2019ZD028 and LZ19C110001).

## Conflict of Interest

The authors declare that the research was conducted in the absence of any commercial or financial relationships that could be construed as a potential conflict of interest.
